# Genome-Wide Analysis of Methylation-Driven Genes and Identification of an Eight-Gene Panel for Prognosis Prediction in Breast Cancer

**DOI:** 10.3389/fgene.2020.00301

**Published:** 2020-04-21

**Authors:** Yanshen Kuang, Ying Wang, Wanli Zhai, Xuning Wang, Bingdong Zhang, Maolin Xu, Shaohua Guo, Mu Ke, Baoqing Jia, Hongyi Liu

**Affiliations:** ^1^Department of General Surgery, The First Medical Center, Chinese PLA General Hospital, Beijing, China; ^2^State Key Laboratory of Membrane Biology, School of Medicine, Tsinghua University, Beijing, China; ^3^Department of General Surgery, Beijing Shijitan Hospital, Capital Medical University, Beijing, China

**Keywords:** epigenetics, DNA methylation, breast cancer, prognosis biomarker, integrative analysis

## Abstract

**Background:**

Aberrant DNA methylation is a crucial epigenetic regulator that is closely related to the occurrence and development of various cancers, including breast cancer (BC). The present study aimed to identify a novel methylation-based prognosis biomarker panel by integrally analyzing gene expression and methylation patterns in BC patients.

**Methods:**

DNA methylation and gene expression data of breast cancer (BRCA) were downloaded from The Cancer Genome Atlas (TCGA). R packages, including ChAMP, SVA, and MethylMix, were applied to identify the unique methylation-driven genes. Subsequently, these genes were subjected to Metascape for GO analysis. Univariant Cox regression was used to identify survival-related genes among the methylation-driven genes. Robust likelihood-based survival modeling was applied to define the prognosis markers. An independent data set (GSE72308) was used for further validation of our risk score system.

**Results:**

A total of 879 DNA methylation-driven genes were identified from 765 BC patients. In the discovery cohort, we identified 50 survival-related methylation-driven genes. Finally, we built an eight-methylation-driven gene panel that serves as prognostic predictors.

**Conclusions:**

Our analysis of transcriptome and methylome variations associated with the survival status of BC patients provides a further understanding of basic biological processes and a basis for the genetic etiology in BC.

## Introduction

Breast cancer (BC), affecting over 1.3 million women globally, is the leading cause of cancer-related death in women (Bray et al., 2018). According to recent statistics, the incidence and mortality of BC have increased rapidly in the past 20 years (Torre et al., 2016). Most current treatments for BC are limited to surgery, radiation, and chemotherapy. Chemoradiotherapy is often accompanied by side effects such as emesis, alopecia, and granulocytopenia, which significantly impair the life quality of patients. Even worse, many BC patients inevitably relapse and metastasize after treatment. With the development of molecular biology and the deepening of oncology research, targeted therapy has become a hotspot in the research of BC treatment, leading the treatment of BC into a personalized and precise era, bringing revolutionary treatment for BC.

The onset of BC is thought to be driven by the accumulation of both genetic and epigenetic alterations (Das and Singal, 2004; Basse and Arock, 2015), the latter corresponding to inheritable gene expression alterations without modification of DNA sequence. Epigenetic alterations are reversible and are more susceptible to environmental factors than genetic alterations. Thus, it is speculated that the epigenetic alterations are mainly involved in the early stages of tumorigenesis. Interestingly, epigenetic alterations are found in early adenomatous polyps (Vymetalkova et al., 2019), supporting their essential role in the early stage of oncogenesis. Epigenetic alterations interfere with gene expression via DNA methylation, post-translational modifications of histones, and miRNA. By repressing tumor suppressors or activating oncogenes, epigenetic modification takes part in the tumorigenesis of BC. DNA methylation on “CpG islands” is the most frequently studied among the various epigenetic modifications. It is well established that hypermethylation of CpG islands in the promoter region of a gene represses its expression (Rauscher et al., 2015). Aberrant DNA methylation is found in various kinds of cancers. As for BC, proapoptotic genes (*HOXA5*, *TMS1*), cell cycle inhibitors (*p16, RASSF1A*), and DNA repair genes (*BRCA* family) are identified as methylation silence genes (Esteller, 2002; Bagadi et al., 2008; Lustberg and Ramaswamy, 2011; Radpour et al., 2011). Previous research has identified the association of DNA methylation and clinicopathological features of BC patients including tumor stage, histological grade, and TP53 status. Furthermore, the methylation of *APC* (Virmani et al., 2001), *CDH1* (Graff et al., 1995), and *CTNNB1* (Suzuki et al., 2008) is found to be closely related to BC development, implying that the progression and prognosis of BC could be influenced by DNA methylation status.

Establishing a robust prognostic risk scoring system might be effective in identifying patients with poor prognosis and guiding the individualized treatment. However, currently there are only few studies focusing on the identification of methylation-based prognosis biomarker panel and the development for a viable prognostic risk scoring system. Therefore, our study aimed to construct a methylation signature prognosis model to provide a further understanding of basic biological processes and a basis for the genetic etiology in BC.

## Materials and Methods

### Data Acquisition

Data of BC patients in the TCGA project (TCGA-BRCA) were downloaded. Only patients with survival information, methylation data, and RNA-seq data were included for further analysis. Sex is the biggest source of variability in methylation data analysis (Aryee et al., 2014), and the variability mainly comes from sex chromosomes (McCarthy et al., 2014). Therefore, probes on sex chromosomes should be removed if a cohort includes patients of both genders. Considering that more than 99% of BC victims are female, we excluded thirteen male patients to maintain the integrity of the data. Finally, 764 tumor samples and 78 solid tissue normal samples were included in our study. Solid tissue normal samples in TCGA database indicate normal tissue samples from individuals with cancer.

Level 1 (Raw data) clinical characteristics data were obtained from TCGA via GDC Xena Hub (Goldman et al., 2019) (Version GDC Release 10.0).

Level 2 (Normalized data) Infinium HumanMethylation450K(HM450K) methylation data (β-value calculations for each probe and sample) were obtained from TCGA via GDC Xena Hub (Version GDC Release 10.0).

Level 3 (Aggregated data) gene expression data were obtained from TCGA via Firehose. In this study, RNA-Seq by Expectation Maximization (RSEM) normalized values were used because RSEM is more accurate compared to FPKM and TPM (Li and Dewey, 2011).

### Data Preprocessing

Preprocessing of gene expression data (RSEM) was conducted by ProcessRNASeqData function in the MethylMix package (version 2.14.0) (Gevaert, 2015; Gevaert et al., 2015; Cedoz et al., 2018), which includes steps as follows: firstly, removing samples and genes with a percentage of NAs greater than 30%; secondly, inputting NAs with the KNN method; thirdly, removing batch effects by Combat adjustment.

**FIGURE 1:**
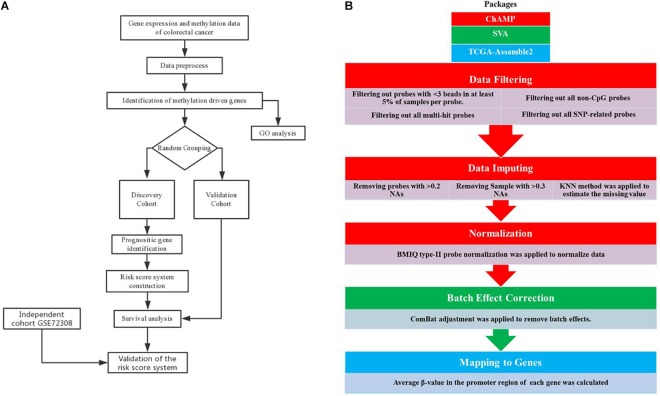
Analysis pipeline. Flowchart of the **(A)** prognosis-scoring model construction process and **(B)** data preprocess.

As is shown in Figure 1B, methylation data preprocess is a five-step procedure involving three R packages. Firstly, we performed data filtering to remove multi-hit probes, probe with <3 beads in at least 5% of the sample per probe, non-CpG probes, and single-nucleotide polymorphism (SNP)-related probes. Secondly, probes with a percentage of NAs greater than 20% and samples with a percentage of NAs greater than 30% were removed, followed by the KNN neighborhood method to estimate the missing value. Thirdly, the processed data were subjected to BMIQ type II probe normalization. The three steps mentioned above were performed by the ChAMP package according to standard protocol (Morris et al., 2014). Then, we use the SVA package (Leek et al., 2012) to remove batch effects using combat algorithms according to standard protocol. Combat algorithms use either parametric or non-parametric empirical Bayes frameworks for adjusting data for batch effects (Johnson et al., 2007). Finally, the average β-value in the promoter region (1500 bp ahead of TSS and 5’UTR) of each gene was calculated using the CalculateSingleValueMethylationData function of TCGA-Assamble2 package (Zhu et al., 2014; Wei et al., 2017).

### Integrative Analysis

We next performed integrative analysis with the MethylMix package. MethylMix integrally analyzes DNA methylation data of normal and cancer samples and the corresponding gene expression data to identify DNA methylation-driven genes. The algorithm of MethylMix includes three steps: the first step identifies transcriptionally predictive methylation; the second step identifies the methylation states of a gene with univariate beta mixture modeling; the third step compares the methylation levels of each methylation state to the mean of DNA methylation level of normal tissue using a Wilcoxon rank-sum test. The main outputs of MethylMix are methylation-related genes, which are both differential methylated and transcriptionally predictive.

### Gene Ontology Analysis of Methylation-Driven Genes

We performed gene ontology (GO) analysis on the methylation-driven genes using the Metascape web-based tool (Zhou et al., 2019). We choose Metascape instead of DAVID because the database of Metascape is updated monthly to ensure that its content is up to date. The Metascape analysis was performed using the default settings.

### Construction of Discovery and Validation Cohort and Identification of Survival-Related Genes

The 764 tumor samples were randomly divided into two cohorts, the discovery cohort and the validation cohort. Chi-square test was applied to compare the distribution of baseline clinical characteristics (age, stage, histological type, etc.) between the two cohorts. Then, each methylation-driven gene was subjected to Kaplan–Meier curve analysis in the discovery cohort. The *p* value was generated by the log-rank test, and genes with *p* < 0.05 were considered survival-related genes. Subsequently, the survival-related genes were used as seed genes for robust likelihood-based survival modeling to screen the genes panel. The screening procedure was according to the standard protocol of R package rbsurv (version 2.42.0). Subsequently, the correlation of these genes was calculated by R package gpairs (version 1.2).

### Construction of Risk Assessment Model and Evaluation

We constructed the regression-coefficients-based risk assessment model by performing multivariate Cox analysis on the discovery cohort. The identified gene panels were subjected to multivariate Cox analysis using the R package rms (version 5.1). After generating the coefficient of each gene, the risk score was generated based on the regression coefficients multiplied by the methylation signature (β value). The risk assessment model was then applied to patients to generate the risk score of each patient. The receiver operating characteristic (ROC) curve was constructed by the pROC package (version 1.15.0) to estimate the prognosis efficiency of the risk assessment model and identify the best threshold (Robin et al., 2011). Subsequently, the samples were divided into the high-risk and low-risk groups by the threshold. Then, Kaplan–Meier analysis was utilized to calculate the overall survival (OS) differences between the high-risk and low-risk groups. Then, multivariate Cox analysis was presented to testify whether the prognosis power of the risk assessment model was independent of other clinical characteristics. We also constructed a nomogram based on the independent prognosis predictor identified by multivariate Cox analysis by the R package rms (version 5.1).

### Validation of the Risk Assessment Model in an Independent Data Set

To validate the prognostic capacity of our risk assessment model, we downloaded the methylation profile and clinical information of GSE72308 (Jeschke et al., 2017) from the NCBI GEO database. Similar methylation and gene expression data preprocessing procedures are conducted. Subsequently, the risk assessment model was applied to GSE72308, with the threshold identified previously.

### Statistical Analysis

R (version 3.6.2) and RStudio (version 1.2.1335) (RStudio Team et al., 2015) were utilized for statistical analysis in this study. Categorical variables were estimated by the chi-square test while continuous variables were estimated by the Student’s *t* test. *p* < 0.05 was considered as statistically significant.

## Results

### Baseline Characters of Patients

The flowchart of the prognosis-scoring model construction process and the data preprocess is shown in Figure 1. To construct and validate a survival prediction model, 764 cancer samples were randomly divided into discovery (*n* = 381) and validation (*n* = 383) cohort. Baseline characteristics were compared between the two cohorts (Table 1). Approximately 70% of BC patients in this study are early stage (stage I and II) patients while 1.57% patients suffer from metastatic carcinoma. The age at diagnosis of these patients ranges from 25 to 90, and the median is 58 years. The histological type of most patients (over 70%) is infiltrating ductal carcinoma (IDC). No significant difference was observed among the baseline characteristics of the two cohorts.

**TABLE 1:** Clinical characteristics of TCGA patients.

Characteristics		Cohort			*p* value
		Discovery	Validation	Total	
Stage	Unknown	5(1.31%)	9(2.34%)	14(1.83%)	0.35
	I	51(13.38%)	65(16.97%)	116(15.18%)	
	II	222(58.26%)	223(58.22%)	445(58.24%)	
	III	96(25.19%)	81(21.14%)	177(23.16%)	
	IV	7(1.83%)	5(1.3%)	12(1.57%)	
Age	≤60	226(59.31%)	204(53.26%)	430(56.28%)	0.092
	>60	155(40.68%)	179(46.73%)	334(43.71%)	
HER2	Unknown	95(24.93%)	99(25.84%)	194(25.39%)	0.755
	Equivocal	59(15.48%)	66(17.23%)	125(16.36%)	
	Intermediate	4(1.04%)	7(1.82%)	11(1.43%)	
	Negative	182(47.76%)	168(43.86%)	350(45.81%)	
	Positive	41(10.76%)	43(11.22%)	84(10.99%)	
ER	Unknown	48(12.59%)	52(13.57%)	100(13.08%)	0.483
	Negative	73(19.16%)	85(22.19%)	158(20.68%)	
	Positive	260(68.24%)	246(64.22%)	506(66.23%)	
PR	Unknown	49(12.86%)	52(13.57%)	101(13.21%)	0.715
	Intermediate	1(0.26%)	1(0.26%)	2(0.26%)	
	Negative	117(30.7%)	103(26.89%)	220(28.79%)	
	Positive	214(56.16%)	227(59.26%)	441(57.72%)	
Histological type	Unknown	1(0.26%)	0(0%)	1(0.13%)	0.411
	Infiltrating ductal carcinoma	267(70.07%)	283(73.89%)	550(71.98%)	
	Infiltrating lobular carcinoma	75(19.68%)	62(16.18%)	137(17.93%)	
	Medullary carcinoma	6(1.57%)	2(0.52%)	8(1.04%)	
	Metaplastic carcinoma	3(0.78%)	2(0.52%)	5(0.65%)	
	Mixed histology	13(3.41%)	10(2.61%)	23(3.01%)	
	Mucinous carcinoma	6(1.57%)	7(1.82%)	13(1.7%)	
	Other specify	10(2.62%)	17(4.43%)	27(3.53%)	
Race	American Indian or Alaska	0(0%)	1(0.26%)	1(0.13%)	0.225
	Asian	15(3.93%)	22(5.74%)	37(4.84%)	
	Black or African American	89(23.35%)	69(18.01%)	158(20.68%)	
	Not reported	6(1.57%)	9(2.34%)	15(1.96%)	
	White	271(71.12%)	282(73.62%)	553(72.38%)	
Radiation therapy	Unknown	34(8.92%)	32(8.35%)	66(8.63%)	0.953
	NO	151(39.63%)	151(39.42%)	302(39.52%)	
	YES	196(51.44%)	200(52.21%)	396(51.83%)	

*No significant difference was found among cohorts.*

### Identification and GO Analysis of Methylation-Driven Genes in BC

**FIGURE 2:**
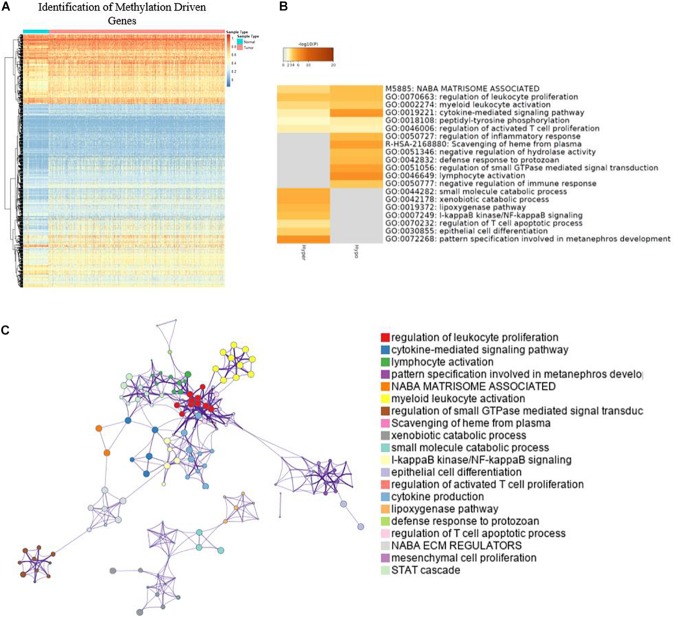
Identification of methylation driven genes and GO analysis. **(A)** Heatmap of global methylation-driven genes in BC primary solid tumor and solid tissue normal. **(B)** Bar graph of enriched terms and methylation-driven genes; only the top 20 clusters were presented. **(C)** Network of enriched terms.

A total of 764 cancer samples and 78 normal samples were included to screen for the methylation-driven genes. MethylMix identified 879 (Figure 2A) methylation-driven genes out of 18,861 genes. For further understanding of functions and metabolic pathways involved for these methylation-driven genes, GO analysis was performed by Metascape. The GO analysis showed that these proteins were involved in various biological processes and the top 20 clusters were presented in Figure 2B. Among them, cytokine-mediated signaling pathway, lymphocyte activation, and pattern specification involved in metanephros development are the most significantly enriched in the function of dual-methylated, hypomethylated, and hypermethylated genes, respectively. The network of the enriched items and the interaction of these genes are shown in Figure 2C. Further, we found that immune-related biological function, including regulation of leukocyte proliferation, myeloid leukocyte activation, and negative regulation of immune response are closely linked to each other, indicating a possible association between DNA methylation and immune responses in BC.

### Identification of Survival-Related Genes

**FIGURE 3:**
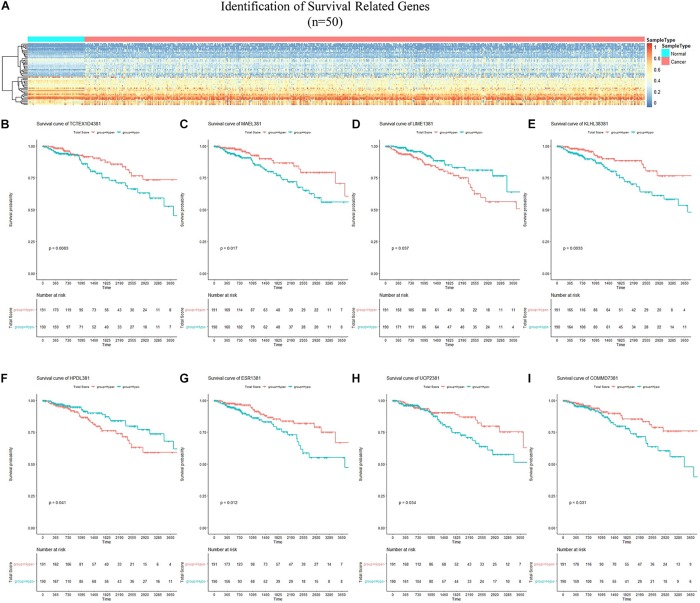
Identification of survival-related genes and generation of the eight-gene panel for the prognosis-scoring model. **(A)** Heatmap of methylation status of 50 survival-related genes identified in the discovery cohort. **(B–I)** Kaplan–Meier curves of the eight genes selected for construction of the prognosis-scoring model. **(B)** TCTEX1D4, **(C)** MALE, **(D)** LIME1, **(E)** KLHL38, **(F)** HPDL, **(G)** ESR1, **(H)** UCP2, **(I)** COMMD7.

**FIGURE 4:**
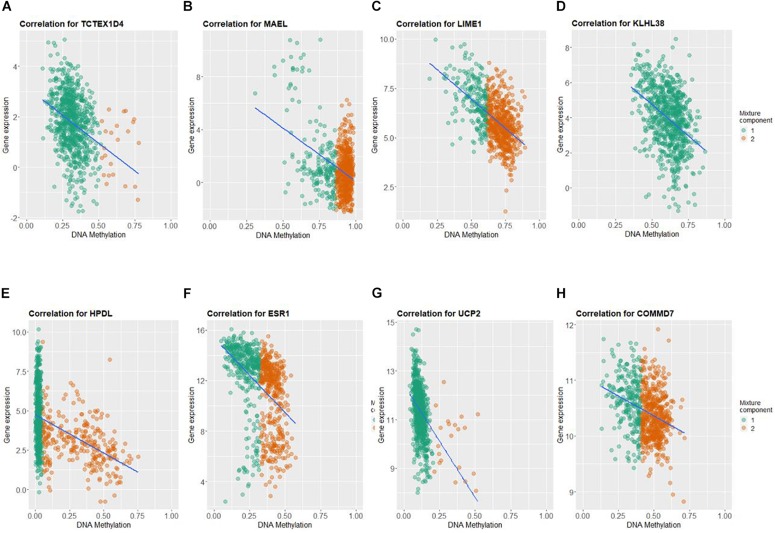
Correlation between DNA methylation and gene expression of the eight genes selected for construction of prognosis-scoring model. **(A)** TCTEX1D4, **(B)** MALE, **(C)** LIME1, **(D)** KLHL38, **(E)** HPDL, **(F)** ESR1, **(G)** UCP2, **(H)** COMMD7. Green dots (component 1): hypomethylated cases; orange dots (component 2): hypermethylated cases.

**FIGURE 5:**
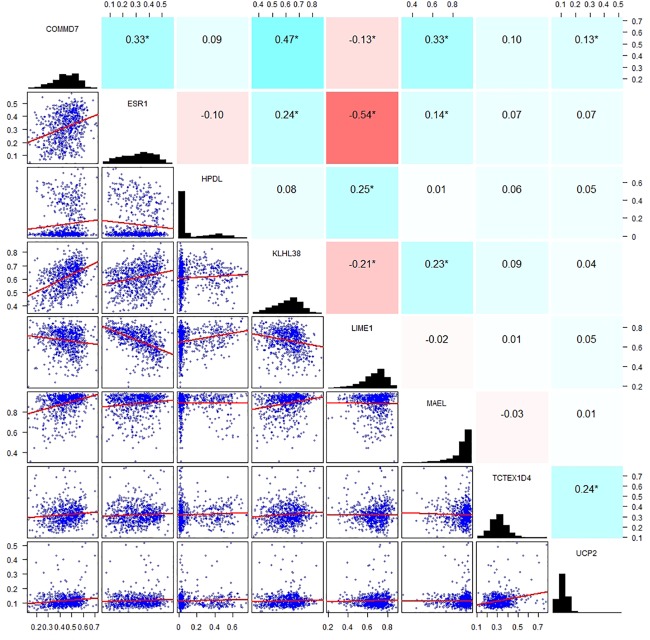
Correlation analysis of the methylation signature of the eight genes. Scatter plots of the methylation signature between genes are presented in the lower left corner, a distribution histogram of methylation signature is shown along the diagonal, correlation coefficients of methylation signature from −1 to +1 is presented in the upper right corner; blue for positive correlation, red for negative correlation, the shade of blue/red represents the strength of correlation between genes.

By applying Kaplan–Meier curve analysis in the discovery cohort, we identified 50 survival-related genes (Figure 3A). By applying the robust likelihood-based survival modeling, eight genes (*TCTEX1D4*, *MALE*, *LIME1*, *KLHL38*, *HPDL*, *ESR1*, *UCP2*, and *COMMD7*) were selected for the construction of the prognostic risk model (Table 2 and Figures 3B–I). The correlation between gene expression and methylation signature was demonstrated in Figure 4 and Supplementary Figure S1. Then, the correlation of the methylation signature of the eight genes was calculated (Figure 5), showing that these genes were not closely related in methylation signature. Moreover, GO analysis of the eight genes showed no interaction, suggesting that there are little redundancy and intersection in the information carried between these genes.

**TABLE 2:** Generation of the eight-gene panel using forward selection (AIC: Akaike information criterion).

Gene	nloglik	AIC	Selected
	261.7	523.39	
COMMD7	258.52	519.04	*
HPDL	254.24	512.49	*
LIME1	253.93	513.86	*
ESR1	253.29	514.58	*
TCTEX1D4	249.37	508.74	*
KLHL38	247.94	507.87	*
MAEL	245.76	505.52	*
UCP2	243.81	503.62	*

### Construction of Methylation Signature-Based Survival Risk Score System

By applying multivariate Cox analysis, an eight-gene methylation signature prognostic risk model was generated as below:

$$\begin{array}{rcl} \mathrm{Risk score} & = & \left(0.190\times COMMD7\right)+\left(1.779\times HPDL\right)\\ & & +\left(1.779\times LIME1\right)-\left(0.87\times ESR1\right)\\ & & -\left(4.07\times TCTEX1D4\right)-\left(2.47\times KLHL38\right)\\ & & -\left(3.03\times MAEL\right)-\left(8.50\times UCP2\;\right) \end{array}$$

**FIGURE 6:**
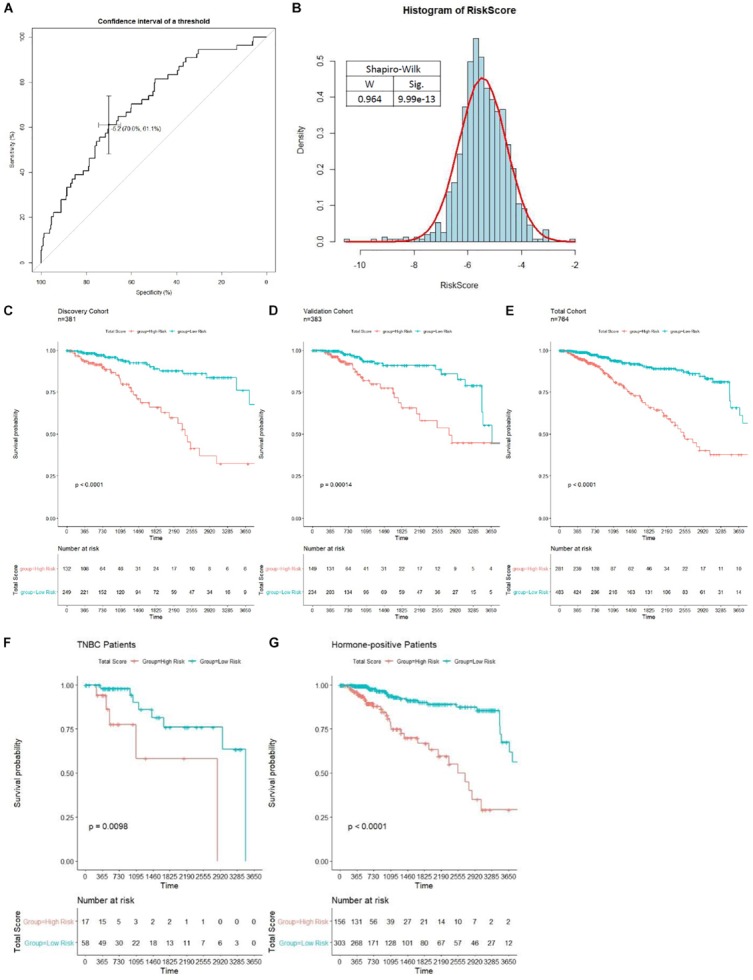
Unsupervised clustering analysis and prognostic analysis based on the prognosis-scoring model clustering. **(A)** Determination of the best cutoff (−5.1737) for clustering patients into high- and low-risk group by ROC curve. **(B)** The distribution of risk score among patients in the total cohort shown in a histogram. Kaplan–Meier curve with log-rank test of patients in discovery **(C)**, validation **(D)**, and total **(E)** cohort were generated to compare the prognosis of high- and low-risk group. Patients in the high-risk group is significantly associated with worse OS in all cohorts (*p* < 0.0001). The prognostic ability of our model in non-TNBC **(F)** and TNBC **(G)** patients is tested by the Kaplan–Meier curve with log-rank test.

Patients in the discovery cohort were subjected to risk score assessment; the best cutoff (−5.174) was determined by the ROC curve (Figure 6A). As is shown in Figure 6B, the distribution of risk score among patients does not obey Gaussian distribution. Patients with risk score of over −5.174 were grouped into the high-risk group. Otherwise, they were grouped into the low-risk group. In the discovery cohort, 132 (34.65%) patients were grouped into the high-risk group, and 249 (65.35%) patients were grouped into the low-risk group. Kaplan–Meier analysis indicated that patients in the high-risk group showed a worse OS, and there was a significant difference (*p* < 0.0001) in prognosis between the two groups (Figure 6C). The median OS of the high-risk and low-risk group was 2,417 and 7,455 days, respectively. Therefore, our results showed that the model we have established could successfully predict the prognosis of BC patients. We then compared the prognostic power between our model and the previously known markers in BC. Unsurprisingly, stage III and IV patients have a worse prognosis than stage I and II (*p* = 0.025), but our model showed a more significant *p* value compared with the pathological grading. It is worth noting that PAM50, ER, HER2, and PR status have no significant impact on patients’ survival (*p* > 0.05, Supplementary Figure S2). Therefore, we demonstrated that our model is superior to the known biomarkers in prognostic prediction of BC.

To further evaluate the prognostic value of our model in various subgroups, we performed survival analysis by Kaplan–Meier plot. The results showed that our model performed well in all subgroups (Figures 6F,G and Supplementary Figure S3). Interestingly, the prognostic ability of our model is significantly weaker in triple-negative breast cancer (TNBC) patients compared to non-TNBC patients (Figures 6F,G). Moreover, our model shows higher power to stratify prognosis in early stage, HER2-negative, and PR-negative patients (Supplementary Figure S3).

**FIGURE 7:**
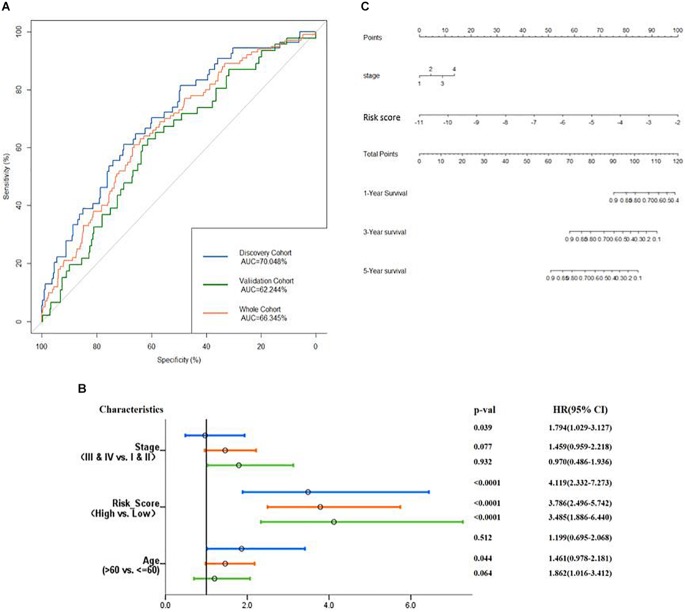
Examine the prognostic power of the prognosis-scoring model. **(A)** AUC of the prognosis-scoring model in each cohort was generated. **(B)** Forest plot of the univariant Cox analysis result shows that our prognosis-scoring model is an independent risk factor aside from histological stage and age. **(C)** Nomogram to predict the 1-, 3-, and 5-year OS.

Then, verification was carried out in a validation cohort by generating the risk score of each patient. With the threshold identified previously (−5.174), 149 (38.9%) patients were grouped into high-risk groups, and 243 (61.1%) patients were grouped into the low-risk group. As expected, patients in the low-risk group had a longer OS and a better prognosis than those in the high-risk group (*p* = 0.00014, Figure 6D). In the total cohort, 281 out of 764 patients were grouped into the high-risk group. Patients with high-risk scores were significantly correlated with poor prognosis (*p* < 0.0001, Figure 6E). Subsequently, to evaluate the power of prognosis prediction of this model, time-dependent ROC analyses were carried out in discovery, validation, and total cohort, and the area of respective ROC curves (AUC) is 0.70048, 0.62244, and 0.66345, respectively (Figure 7A). This result showed that our eight-gene methylation risk score system performed well in stratifying patients into high-risk and low-risk groups.

The total cohort was then subjected to multivariate Cox analysis. As shown in Figure 7B, the risk score is an independent prognostic factor in the discovery, validation, and total cohort (*p* value <0.001). Considering only stage has a significant impact on patients’ survival (Supplementary Figure S2), our nomogram (Figure 7C) contains two variables, stage and risk score.

### Validation of the Survival Risk Score System in an Independent Data Set

**FIGURE 8:**
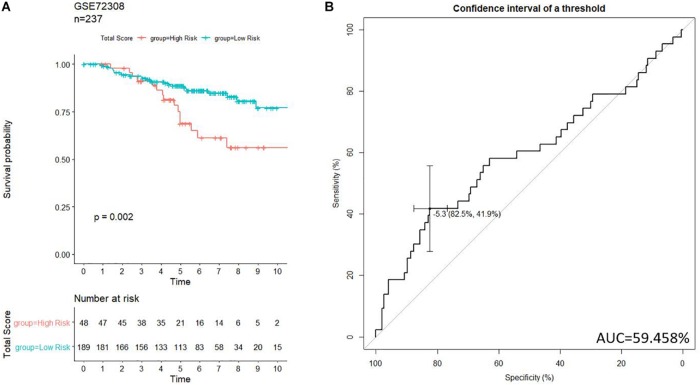
Validation of the prognosis-scoring model in an independent data set (GSE72308). **(A)** Our model successfully clustered patients into high- and low-risk group, and patients in the high-risk group are significantly associated with worse prognosis. **(B)** ROC curve was generated to test.

To further examine the prognostic values of our risk score system, an independent data set (GSE72308, *n* = 237) was downloaded from GEO. The data set was subject to our risk model for generating the risk score of each patient. Patients in GSE72308 were grouped in high risk (*n* = 48, 20.3%) and low risk (*n* = 189, 79.7%) by the threshold identified previously. Kaplan–Meier curve (Figure 8A) indicated similar trends to the TCGA patients. ROC curve (Figure 8B) was generated to evaluate the capacity of the prognosis prediction of the risk score system. The prognosis of high-risk patients was significantly worse than that of low-risk patients. As shown in Figure 8B, the best threshold identified in the GSE72308 data set was −5.256, very close to the threshold of the discovery cohort (−5.174), implying the stability of our model.

## Discussion

Breast cancer is a heterogeneous disease with various therapeutic responses and outcomes. Apart from surgical treatment, chemotherapy and target therapy are essential for BC treatment. However, growing therapeutic options require an accurate classification system to guide personalized treatment. Traditionally, BC is staged by histopathological criteria including size, level of invasiveness and lymph node infiltration, and well-established biomarkers, including ER, PR, and HER2. However, the prognostic predicting power of conventional BC staging system prognosis is not satisfying. Recently, the study of identifying gene-based methylation signature prognostic model for BC has attracted much attention, and several papers have reported the feasibility of this method (Bao et al., 2019; Chen et al., 2019; Du et al., 2019; Qi et al., 2019). In the present study, integrated analysis on patients in the BRCA project of TCGA was carried out by MethylMix, identifying 879 methylation-driven genes with different methylation patterns in tumor and normal tissues. Our GO term analysis revealed that the differentially expressed DNA methylation-driven genes were involved in cytokine-mediated signaling pathway and lymphocyte activation. Network analysis of these genes showed a strong relation to immuno-regulation, suggesting a close relationship between DNA methylation and immunology. Moreover, we constructed an eight-methylation-driven gene panel that serves as prognostic predictors to identify high-risk and low-risk patients, providing a guide for personalized therapy. Compared with a previous study, our model outputs a risk score and performed further verification with a validation cohort and an independent data set, which makes our proposed model more reliable than the genes and models in other articles. However, it has better prognostic power in early stage, HER2-negative, and PR-negative patients, respectively. Considering the uneven distribution of the number of cases between subgroups, we speculate that the difference is caused by the instability of the limited subgroup cases. Our model shows lower power to stratify prognosis in TNBC patients for several reasons. Firstly, the number of TNBC cases is limited. Secondly, biological characteristics of TNBC, including methylation pattern, are very unique, which requires further investigation. Finally, the prognosis of TNBC patients is worse than that of non-TNBC patients. The management of TNBC patients is a hotspot in BC research, and a prognosis prediction model for TNBC patients has great potential. We also demonstrated that our model is superior to the known biomarkers in prognostic prediction of BC. The WGBS and EPIC array covers more genomic regions compared to the HM450k (Pidsley et al., 2016). We used HM450k array data in this study because large population data of WGBS or EPIC array are not available yet. Moreover, further testing of our model on different platforms is needed in the future. Besides, a prospective study is needed to further test the reliability of our model.

Our model involves the methylation signature of eight genes. Among them, ESR1, encoding estrogen receptor-alpha (ER-α), is the most investigated gene in BC. ERα-positive BCs account for 70–80% of all BC types (Holst et al., 2007). It is a hormone-dependent tumor, and stimulation with long-term estrogen increases the risk of cancer recurrence and metastasis. Therefore, endocrine therapy or other targeted therapies for ERα and its signaling pathways are essential components of comprehensive treatment for ER-positive BC patients. It is reported that COMMD7 plays a role in a novel NF-κB-positive feedback loop by dual-directional regulation in hepatocellular carcinoma (HCC) (Esposito et al., 2016). The function of TCTEX1D4 is not thoroughly investigated. Currently, it is known as a protein phosphatase one interactor (Korrodi-Gregório et al., 2013). He et al. (2016) identified Kelch-like protein 24 (KLHL24) as a maintainer of skin integrity by balancing degradation and intermediate filament stability. MAELSTROM protein (MAEL) is a novel diagnostic biomarker for gastric cancer. A previous study revealed that the function of MAEL is closely linked to epithelial–mesenchymal transition (EMT) and stem cell properties (Zhang et al., 2017). Lck Interacting Transmembrane Adaptor 1 (LIME1) is a transmembrane adaptor involved in the activation of BCR (B-cell antigen receptor)-mediated signaling via interaction with Lck and Lyn (Hořejšì, 2004). However, to our knowledge, the function of 4-Hydroxyphenylpyruvate Dioxygenase Like (HPDL) remains unclear. Even though the prognostic impact of these genes is identified in our study, their function, except for ESR1, is generally unclear in BC. Therefore, the biological function of these genes requires further investigation.

## Conclusion

In this study, we performed an integrated analysis to identify an eight-gene DNA methylation score system that is prognostically associated with the BRCA project of the TCGA database and an independent data set (GSE72308). The scoring system could distinguish between high-risk and low-risk patients for guiding individualized treatment. Moreover, it might provide novel potential therapeutic targets for BC. However, our score system requires further validation by a prospective study in the future.

## Data Availability Statement

The data sets generated and analyzed during the current study are publicly available in the TCGA repository (https://portal.gdc.cancer.gov/) and GEO database (under accession code: GSE72308).

## Ethics Statement

This study is a secondary data analysis. The data used in this study were collected as part of a clinical trial or medical records. The institutional and/or national research ethics committee has approved the data collection and management process.

## Author Contributions

YK designed the model and the computational framework and analyzed the data. YK, BZ, XW, MX, SG, and MK carried out the implementation. YK, YW, and WZ wrote the manuscript with input from all authors. HL and BJ conceived the study and were in charge of overall direction and planning.

## Conflict of Interest

The authors declare that the research was conducted in the absence of any commercial or financial relationships that could be construed as a potential conflict of interest.

## Abbreviations

AUC: area under the receiver operating characteristic curve
BC: breast cancer
CIMP: CpG methylation profiles
ER: estrogen receptor
GO: Gene ontology
HCC: hepatocellular carcinoma.
ICB: immune-checkpoint blockade
OS: overall survival
ROC: receiver operating characteristic
RSEM: RNA-Seq by expectation maximization
SNP: single-nucleotide polymorphism
TCGA: The Cancer Genome Atlas
WGBS: whole-genome bisulfite sequencing.
