# Supplementary material for: Genome-Wide Analysis of Methylation-Driven Genes and Identification of an Eight-Gene Panel for Prognosis Prediction in Breast Cancer
Source: Front Genet. 2020 Apr 21;11:301. doi: 10.3389/fgene.2020.00301 (PMC7186397; doi:10.3389/fgene.2020.00301)
Supplement: Supplementary file 1 [file Presentation_1.PPTX]

## Slide 1
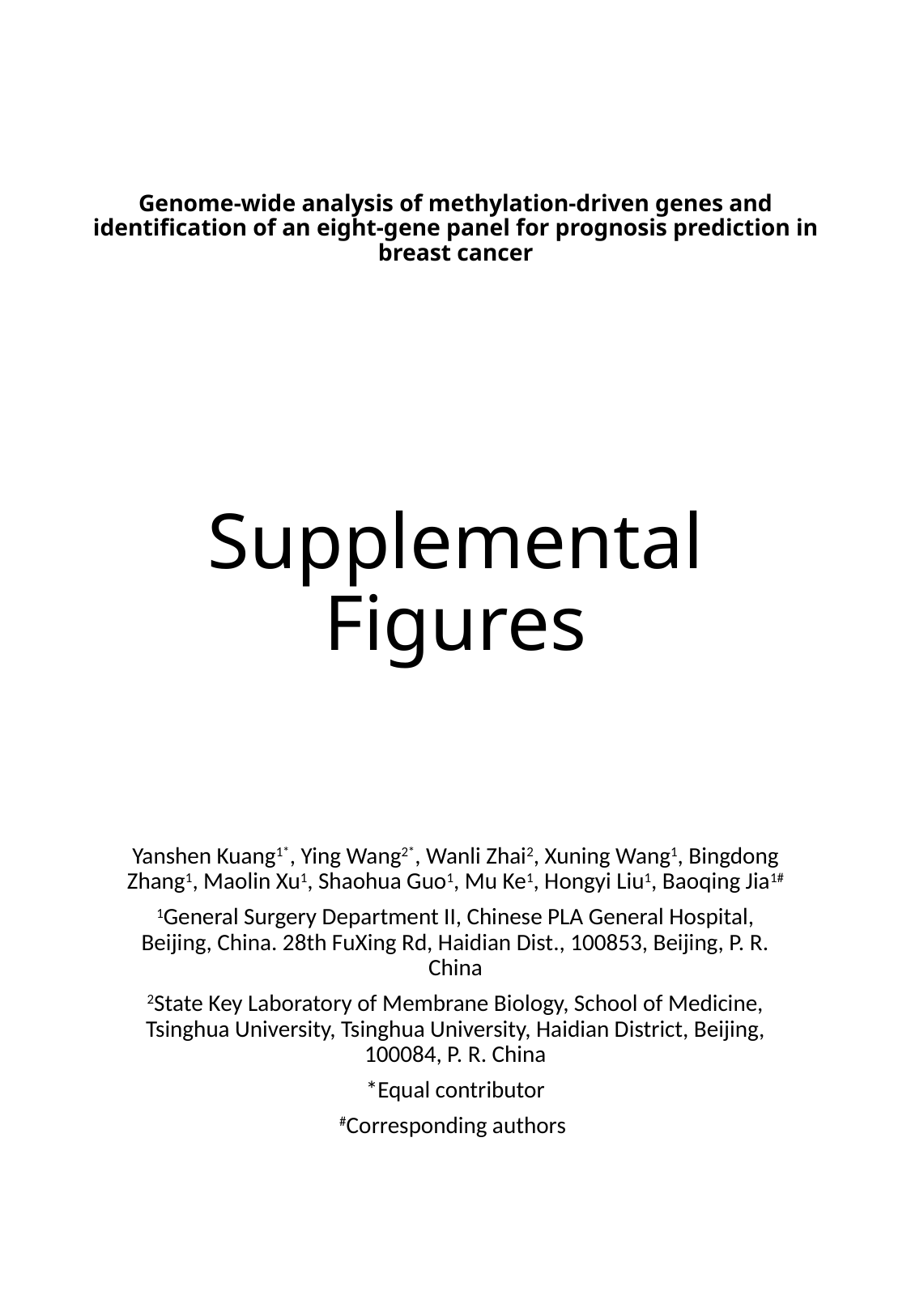

Genome-wide analysis of methylation-driven genes and identification of an eight-gene panel for prognosis prediction in breast cancer
# Supplemental Figures
Yanshen Kuang1*, Ying Wang2*, Wanli Zhai2, Xuning Wang1, Bingdong Zhang1, Maolin Xu1, Shaohua Guo1, Mu Ke1, Hongyi Liu1, Baoqing Jia1#
1General Surgery Department II, Chinese PLA General Hospital, Beijing, China. 28th FuXing Rd, Haidian Dist., 100853, Beijing, P. R. China
2State Key Laboratory of Membrane Biology, School of Medicine, Tsinghua University, Tsinghua University, Haidian District, Beijing, 100084, P. R. China
*Equal contributor
#Corresponding authors

## Slide 2
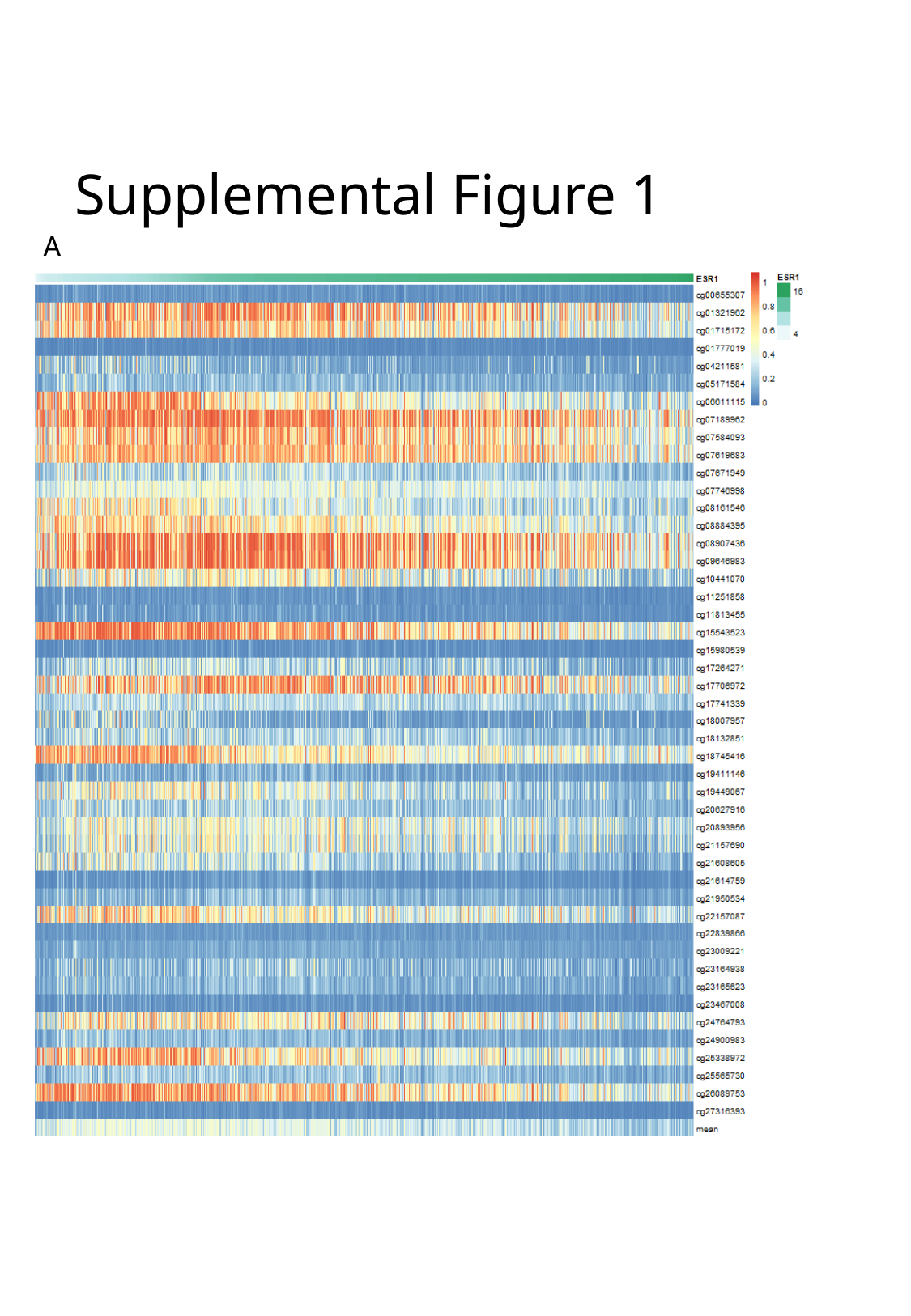

# Supplemental Figure 1
A

## Slide 3
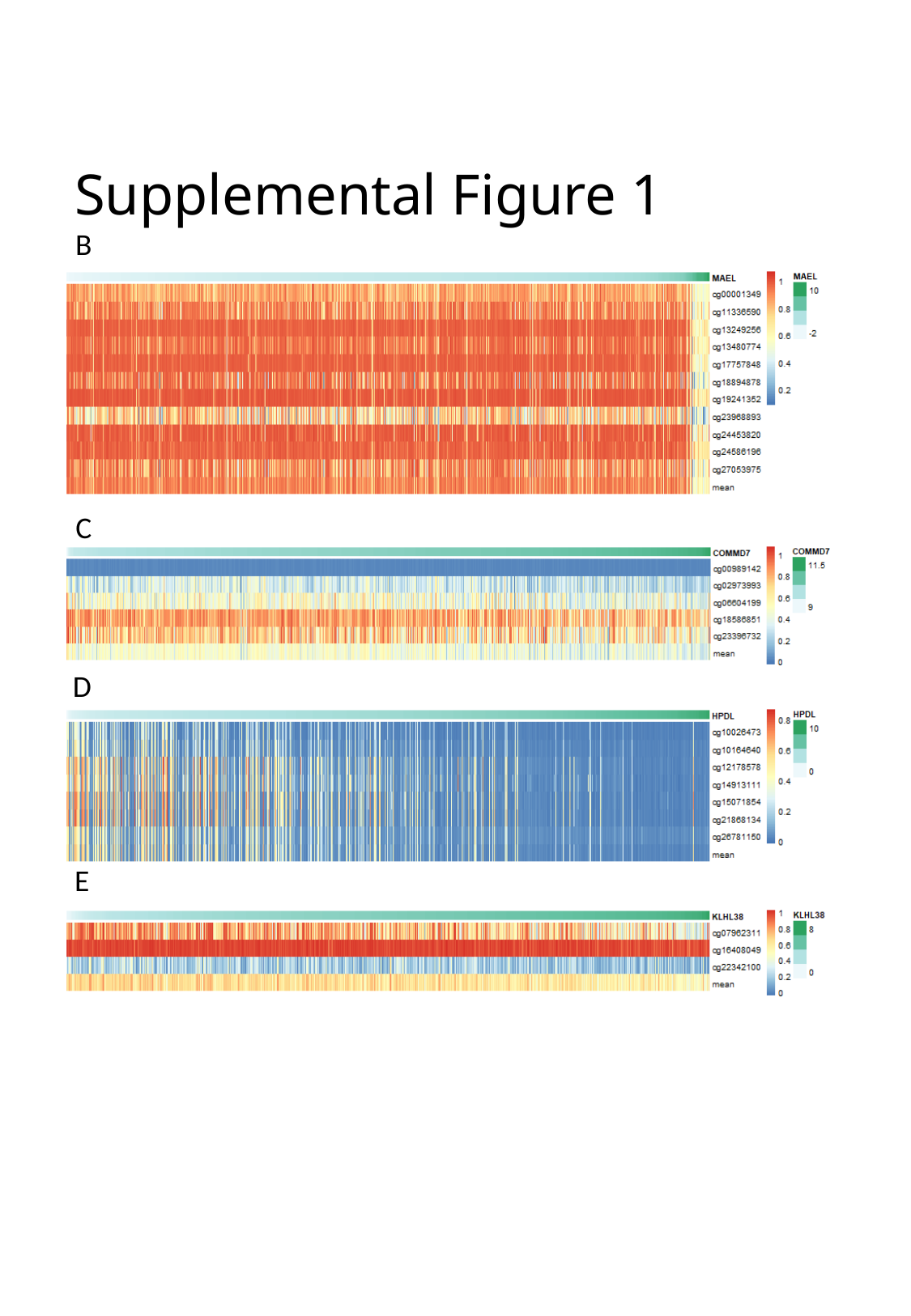

# Supplemental Figure 1
B
C
D
E

## Slide 4
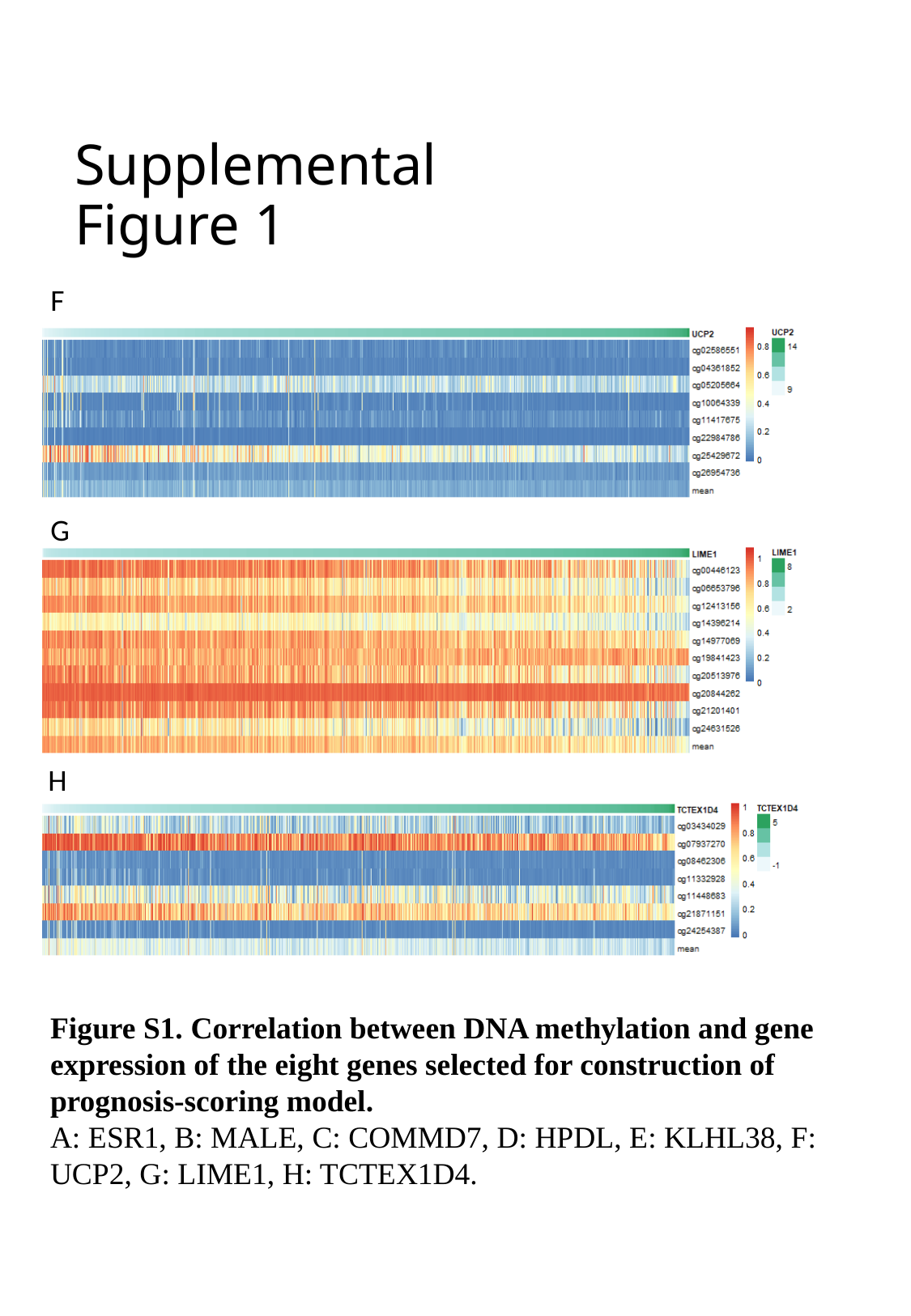

# Supplemental Figure 1
F
G
H
Figure S1. Correlation between DNA methylation and gene expression of the eight genes selected for construction of prognosis-scoring model.
A: ESR1, B: MALE, C: COMMD7, D: HPDL, E: KLHL38, F: UCP2, G: LIME1, H: TCTEX1D4.

## Slide 5
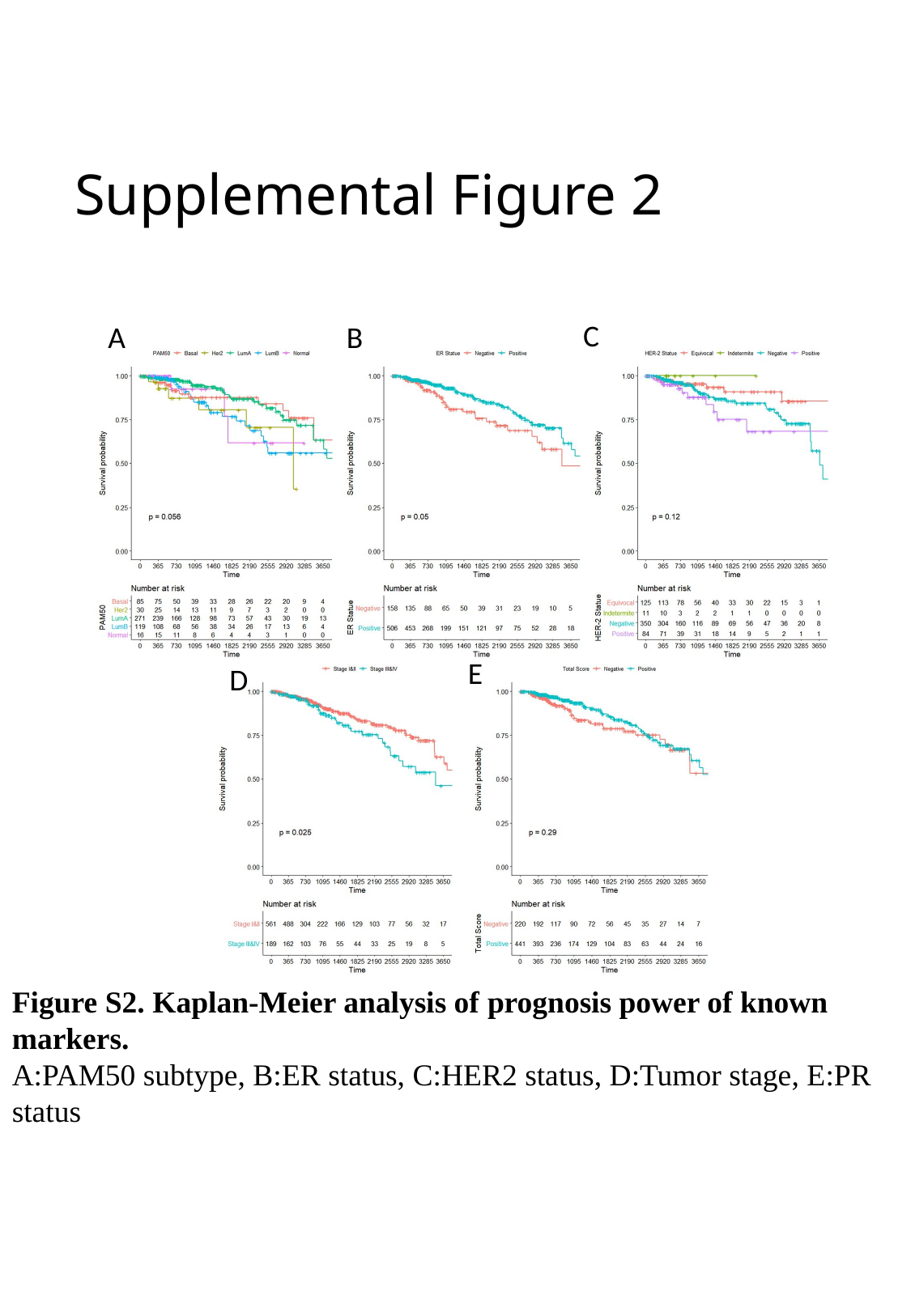

# Supplemental Figure 2
C
A
B
E
D
Figure S2. Kaplan-Meier analysis of prognosis power of known markers.
A:PAM50 subtype, B:ER status, C:HER2 status, D:Tumor stage, E:PR status

## Slide 6
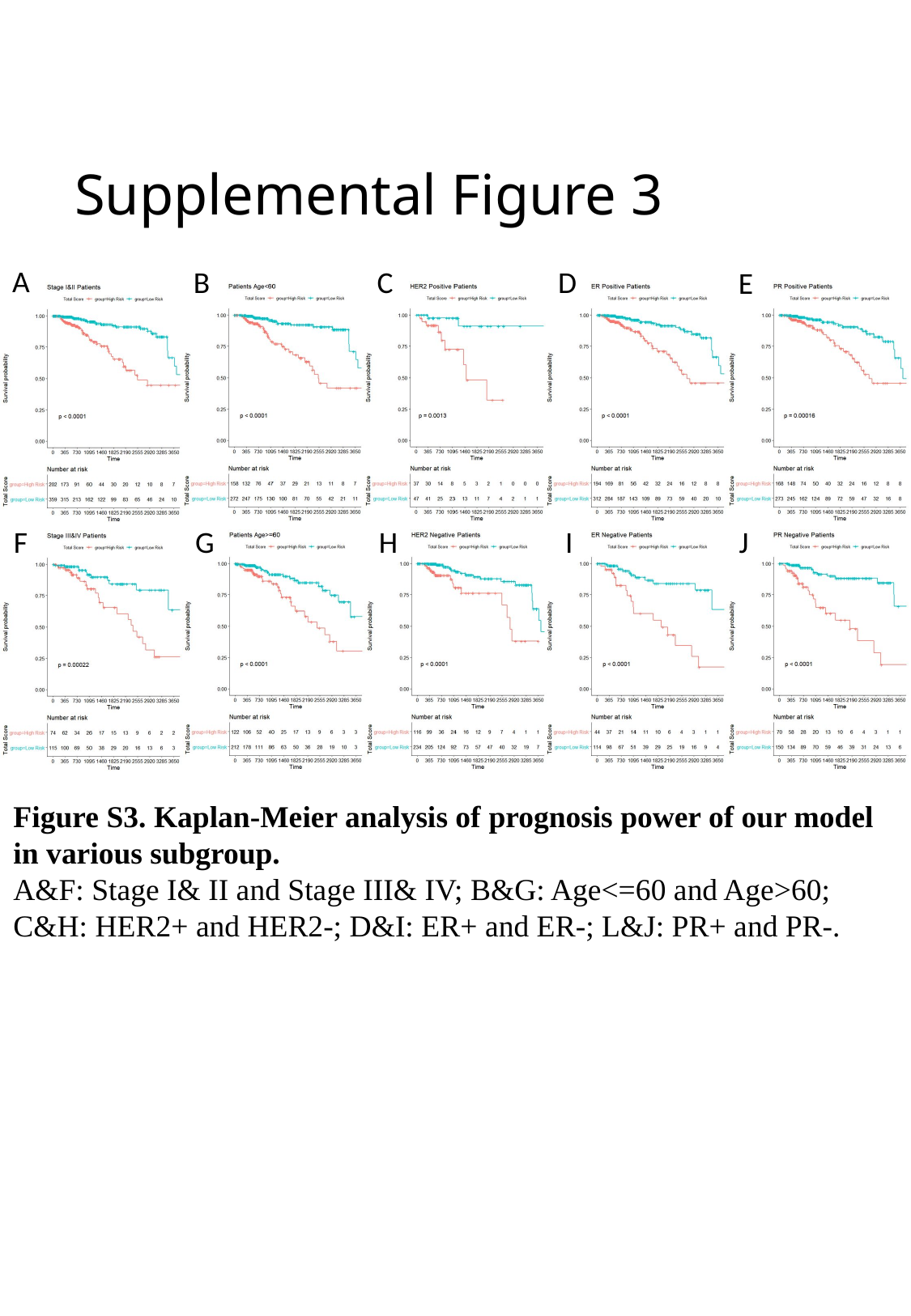

# Supplemental Figure 3
A
B
C
D
E
I
J
G
H
F
Figure S3. Kaplan-Meier analysis of prognosis power of our model in various subgroup.
A&F: Stage I& II and Stage III& IV; B&G: Age<=60 and Age>60; C&H: HER2+ and HER2-; D&I: ER+ and ER-; L&J: PR+ and PR-.
